# A Human-Centered Approach for a Student Mental Health and Well-Being Mobile App: Protocol for Development, Implementation, and Evaluation

**DOI:** 10.2196/68368

**Published:** 2025-07-18

**Authors:** Maryam Gholami, David Wing, Manas Satish Bedmutha, Job Godino, Anahi Ibarra, Byron Fergerson, Nicole May, Christopher A Longhurst, Nadir Weibel, Anne Duffy, Heidi Rataj, Karandeep Singh, Kevin Patrick

**Affiliations:** 1 Altman Clinical and Translational Research Institute University of California San Diego Health La Jolla, CA United States; 2 Herbert Wertheim School of Public Health and Human Longevity Science University of California San Diego La Jolla, CA United States; 3 Exercise and Physical Activity Resource Center University of California, San Diego La Jolla, CA United States; 4 Computer Science and Engineering University of California San Diego La Jolla, CA United States; 5 School of Medicine University of California San Diego Health La Jolla, CA United States; 6 Joan and Irwin Jacobs Center for Health Innovation University of California San Diego Health La Jolla, CA United States; 7 Department of Psychiatry Queen’s University Kingston, ON Canada; 8 Department of Psychiatry University of Oxford Oxford United Kingdom

**Keywords:** student-centered, well-being application, student mental health, student wellness, university well-being

## Abstract

**Background:**

The rising prevalence of mental health concerns among students is prompting universities to explore innovative solutions to support student well-being. This paper describes the protocol for the development, implementation, and evaluation of a mobile app designed to address the mental health and wellness needs of students. This project employs a student-centered approach, partnering with students from the initial needs analysis through to the final design and implementation stages.

**Objective:**

The app aims to increase the use of campus resources that address student mental health and wellness by improving the awareness of these resources through user-designated preferences that are established on the initial use of the app and then iteratively refined as it is used. The app is linked to the campus student’s electronic health record so that health and wellness services can be coordinated and enhanced and the student journey to and through care become more seamless. The long-term objective is to leverage data from both the app and electronic health record to improve individual and population health for the entire campus.

**Methods:**

At the beginning of the project, a comprehensive logic model was created to outline the core inputs, activities, outputs, outcomes, and long-term impacts that were desired for the app. The model emphasized the integration of the app within existing campus mental health and wellness services and its potential to foster a culture of well-being across the university community. An evaluation plan was developed that incorporates both quantitative and qualitative methods through biannual assessments to track trends and app impact across campus in addition to feasibility, acceptability, and usability as well as its reach, effectiveness, and sustainability. Validated measures such as the Patient Health Questionnaire and Generalized Anxiety Disorder scale were selected to track changes in mental health and wellness, while custom surveys and analytics will gauge user engagement and satisfaction. New students, including freshmen, transfers, and first-year medical students, are invited to participate after giving informed consent. They receive compensation for their involvement in both quantitative and qualitative assessments.

**Results:**

As of March 2025, we have collected over 600 survey responses from freshmen, transfer, and medical students. A second survey round and additional focus groups are planned for April to May 2025. No analyses have been conducted yet. The findings from this project have the potential to inform similar efforts at other institutions and contribute to the broader field of digital mental health innovation and the development of well-being interventions tailored for young people.

**Conclusions:**

By leveraging digital technology and actively engaging students in supporting their well-being, this initiative represents an innovative user-centered approach to improve mental health and wellness support on university campuses.

**International Registered Report Identifier (IRRID):**

DERR1-10.2196/68368

## Introduction

Students in higher education are tasked with navigating a major life transition that can be incredibly challenging as they move away from home, manage higher academic demands, navigate new social environments, and take on more personal responsibilities for their daily lives [1,2]. These changes can lead to high levels of stress and the onset or exacerbation of mental health issues [3].

Research has shown that university students experience high rates of mental health concerns and reduced well-being. In a 2024 National College Health Assessment survey of 103,639 students at 154 institutions, over 20% of the undergraduate and 15% of the graduate student respondents reported experiencing serious psychological distress. Anxiety and depression are the most prevalent problems, with over 35% of the undergraduate and 33% of the graduate students reporting having anxiety, and over 26% of the undergraduate and 25% of the graduate students reporting depression as chronic conditions [4]. Other studies globally have reported similar rates of common mental health concerns among university students, including anxiety, depression, eating disorders, substance abuse, and self-harm/suicidal ideation [5-8]. Further, there is evidence that the COVID-19 pandemic and associated social restrictions and campus closures amplified these rates [9].

Concerningly, many students who are struggling do not seek help. Estimates suggest that only around 30%-40% of the university students with mental health needs actually receive treatment [7,10]. There are many barriers to accessing mental health and wellness services, including stigma, lack of awareness of available resources, financial constraints, and difficulty navigating the complex health care systems [11]. Even for those who do seek help, mental health and wellness resources in many university campuses are often overburdened and insufficient to meet student needs [12]. Counseling centers frequently report long waitlists, limited session caps, and inadequate funding and staffing [13]. These resource constraints make it challenging for counseling centers to provide high-quality, comprehensive care.

Addressing the mental health and well-being crisis on university campuses requires a multifaceted approach [14]. In addition to increasing staffing for on-campus counseling, colleges should also focus on implementing evidence-based prevention and early intervention programs. This could include initiatives to reduce stigma, educate students on mental health and wellness, and teach coping strategies [15]. Strengthening connections between campus mental health and wellness services and local health care providers in the community can also help expand care available to students. Digital technologies embedded into care pathways is a recognized sustainable way forward to build capacity for student well-being and mental health support and improve transition to and through support resources and services.

Ultimately, addressing student mental health and well-being is crucial not only for individual well-being but also for academic success and long-term outcomes [16]. By taking steps to better support the mental health and wellness needs of university students, institutions can foster healthier and more resilient campus communities.

In recognition of the growing mental health and wellness crisis among university students, many institutions of higher education have implemented programs and initiatives aimed at supporting student well-being. These efforts have ranged from expanding on-campus counseling services to implementing mental health and wellness education and prevention initiatives. One approach has been to increase the resources and staff available at university counseling centers. Many universities have hired additional counselors, psychologists, and psychiatrists to expand their clinical capacity and reduce long waitlists [13]. Some have also extended hours of operation or established satellite locations to improve accessibility. However, the demand for services often continues to outpace available resources, limiting the impact of these expansions. Moreover, students who utilize on-campus counseling and wellness services have improved mental health and well-being and academic outcomes, but the overall impact on the broader campus population has been more limited, with persistent unmet needs and disparities in both utilizing the available services and access to care [17].

Colleges have also worked to destigmatize mental health issues and encourage students to seek help when needed. Awareness campaigns, gatekeeper trainings, and peer-to-peer support programs have been employed to foster a more open dialogue around mental health on campus [7]. Although these initiatives have shown some promise in reducing stigma, evidences of their effects on the actual utilization of services have been mixed.

Beyond clinical services, many institutions have implemented upstream prevention and early intervention programs. These have included efforts to promote stress management, healthy coping strategies, and emotional regulation skills through workshops, courses, and web-based modules [15,18]. Some colleges integrate mental health and wellness screenings into routine health checkups to facilitate early identification and treatment of issues, but there is limited published evidence of the effectiveness of these initiatives.

Moving forward, a comprehensive, public health-informed approach will likely be needed to truly address the mental health and wellness crisis affecting university students. This could involve better integrating campus mental health and wellness services with on and off-campus health care providers, expanding preventive programming, and working to change campus cultures and norms around mental health and wellness. Sustained institutional commitment and investment will be crucial to the success of these efforts.

In recent years, approaches that link digital mental health interventions with wellness services have emerged as a promising approach to expand access and use of support services for university students [19-21]. These web-based and app-based tools offer a variety of self-guided well-being interventions, teletherapy services, and mental health education resources that can complement traditional in-person counseling [22].

The rapid growth of digital solutions has been driven in part by their potential to overcome key barriers that prevent students from seeking help, such as perceived stigma, time constraints, and transportation challenges [23]. Many digital platforms allow users to access services discreetly and conveniently from their own devices [24]. Studies have also found that students may be more willing to engage with mental health and wellness support delivered via technology compared to traditional in-person therapy [21].

Evaluations of digital mental health interventions and well-being programs geared toward university populations have generally found positive outcomes. For example, a meta-analysis of internet-based cognitive behavioral therapy interventions for anxiety among university students showed significant reductions in symptom severity compared to control groups [18]. Other research has linked the use of mental health and wellness apps to improve symptoms of depression, stress, and disordered eating among students [25].

However, the success of digital intervention tools has been tempered by some important challenges. Uptake and sustained engagement with these programs is often low, with many students downloading apps but then failing to use them regularly [24,26]. Concerns about data privacy and security with digital platforms limit the appeal for some users, and the recent proliferation of mental health and wellness apps has resulted in a crowded and unregulated market, making it difficult for students to identify high-quality, evidence-based options. Finally, the lack of interfaces that enable data exchange between these tools and campus health and counseling services hinders the ability to provide a continuum of care for students [21].

Moreover, Patrick et al [27] argue that traditional research methods and approaches to digital health interventions overall are inadequate for the increasingly data-rich environment of digital health. They propose an “agile science” approach that emphasizes rapid iteration, ongoing computational modeling, and iterative personalization that dynamically address the complexity inherent in human existence. This approach allows researchers and interventionists to move beyond static, descriptive theories toward dynamic, predictive models that can drive more effective and sustainable digital health solutions.

Therefore, moving forward, a more strategic and coordinated approach is needed that integrates digital mental health innovation with health and wellness services on university campuses to maximize the impact and more holistically address the well-being needs of student. To accomplish this, in January 2023, the Joan and Irwin Jacobs Center for Health Innovation at University of California San Diego (UCSD) Health, in partnership with UCSD’s Campus Health and Wellness program, began to develop an app and the supporting information technology platform, branded as Willo. This paper describes the approach taken to develop Willo and then focuses on the plan for implementation and evaluation.

## Methods

This protocol is reported in accordance with the SPIRIT (Standard Protocol Items: Recommendations for Interventional Trials) 2013 statement [28] (Multimedia Appendix 1).

### User-Centered Design Process

From its inception, the development of Willo was in co-partnership with students, recognizing that students would be their primary users and beneficiaries. The project team collaborated with students throughout the design process to ensure that the final product would be tailored to their specific needs, preferences, and challenges.

The process began with a comprehensive gap analysis, where students from diverse backgrounds were invited to participate in focus groups and individual interviews (Multimedia Appendix 2). These sessions explored students’ mental health and wellness needs, the challenges they face in accessing support, and the gaps they perceive in existing campus services. Students shared their experiences managing stress, symptoms of anxiety and depression, along with other wellness concerns, providing valuable insights into the priority need areas for additional support and informing and shaping the app’s features and overall value proposition.

As the design process progressed, students continued to play an active central role in refining the app’s features and functionality. Through surveys, usability testing sessions, and design workshops, students provided feedback on various prototypes and concepts. They helped prioritize the features that would be the most useful and likely to be used regularly, such as mood tracking, guided meditation exercises, or anonymous peer-support forums. Students also offered insights into potential barriers to app usage, such as concerns about privacy or stigma, which informed the development of user-friendly interfaces and confidentiality measures. By maintaining this student-centered approach throughout the design process, the team ensured that the resulting app would be not only evidence-based by using state-of-the-art privacy and technological features and evidence-based content but highly relevant and appealing to its intended user base. This student-centered methodology fostered trust and transparency while enabling rapid, iterative refinement of the platform through continuous end-user feedback and contributions.

### Design Process

**Figure 1:**
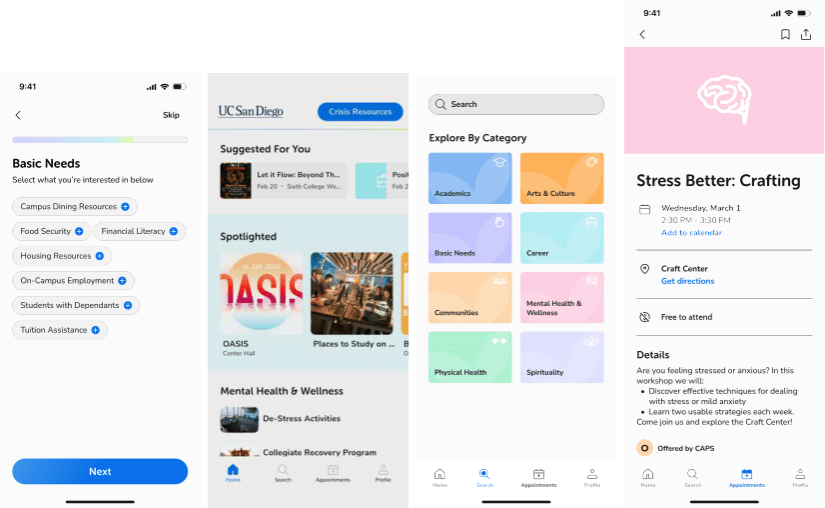
Willo app. From left to right: onboarding interface, main home screen, search and exploration interface, and a detailed view of an individual event or resource.

We conducted iterative co-design studies with students and key stakeholders involved in student well-being, including care providers, clinical staff, and university administrators, to identify the critical challenges affecting student well-being. A recurrent theme across these sessions was the absence of a personalized, regularly updated medium through which students could effectively engage with available university resources. Although a range of services existed, participants emphasized the need for an integrated system to support continuous search, discovery, and engagement. In response, we conceptualized a resource aggregator that could centralize both static resources (eg, physical centers, institutional services) and dynamic offerings (eg, events) organized by the core dimensions of wellness and relevant thematic tags. These tags served as the foundation for tailoring resource recommendations to individual student needs and preferences. Figure 1 illustrates the Willow app interface screens.

### Logic Model

**Figure 2:**
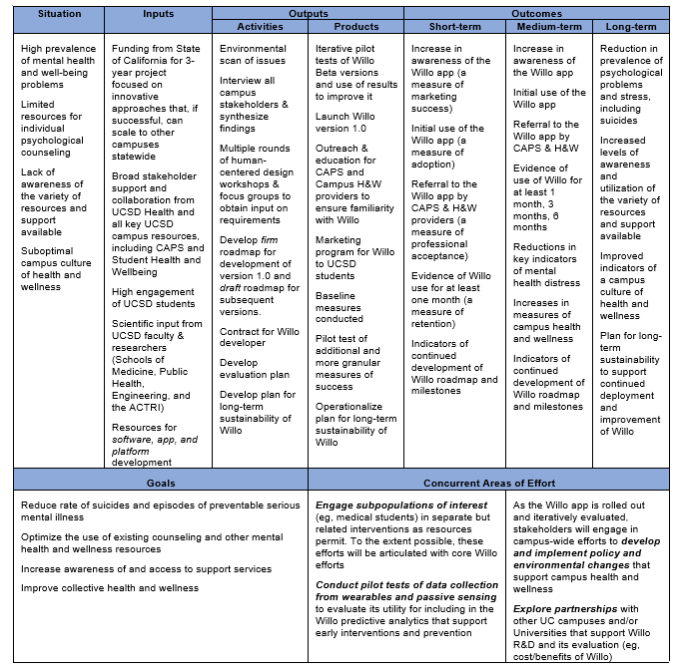
Logic model. ACTRI: Altman Clinical and Translational Research Institute; CAPS: Counseling and Psychological Services; H&W: Health and Wellness; R&D: Research and Development; UC: University of California; UCSD: University of California San Diego.

Based on the best practices in program evaluation in public health [29], our process of evaluation began with the development of a logic model that established the key inputs, activities, outputs, and outcomes associated with the implementation and evaluation of our student mental health and wellness mobile app. A scientific advisory committee composed of students with lived experience, experts in student health and well-being, medicine, psychology, digital interventions, public health, and health services evaluation, was formed to provide input to the app development team on each component of the model and to recommend measurable outcomes for each that would help determine the effectiveness and feasibility of the app. The logic model highlights the multifaceted approach needed to drive positive impacts at both the individual and institutional level. The short, intermediate, and long-term outcomes reflect the iterative and sustained nature of effecting meaningful change in campus mental health and well-being (Figure 2).

The advisory committee also made recommendations about the resources and assets necessary for the successful implementation of the project. These inputs included funding from university administration and external grants and interdisciplinary stakeholders, including clinical psychologists, student health clinicians, computer and data scientists, and user experience designers. Further partnerships with campus counseling centers and other student support services and access to university student population and data were identified as critical.

The essential actions and processes that have been undertaken to achieve the project’s goals are to conduct user research and needs assessment with university students, design and develop a mobile app prototype with evidence-based features, implement pilot testing and iterative improvements based on user feedback, and promote app availability and usage through campus-wide marketing and education.

The immediate, tangible output of the project activities will include a fully functional mobile app for student mental health and wellness support, an increased awareness and utilization of campus mental health and well-being resources, and outreach and education for campus service providers to familiarize them with the app. The committee made recommendations about the following short-term, intermediate, and long-term changes to be assessed as a result of the project and how these lead to broader systemic change.

1. The short-term outcomes include improved mental health and wellness literacy and help-seeking attitudes among students, increased engagement with evidence-based self-management strategies, and enhanced feelings of social connectedness and reduced isolation.
2. Intermediate outcomes include reduced symptoms of anxiety, depression, and other common mental health issues and increased utilization of the app and on-campus counseling and support services.
3. Long-term outcomes are expected to be a positive shift in the campus culture around mental health and well-being; sustainable adoption and integration of the app through students; and improved overall student health, satisfaction, and success outcomes.
4. The broader systemic changes that the project aims to contribute to over time would be a healthier and more resilient university student population, leading to reduced mental health–related costs and productivity losses for universities.

The committee emphasized the importance of viewing this logic model as a dynamic framework, subject to refinement and adaptation, as new evidence emerges and the project evolves. They also stressed the need for rigorous evaluation at each stage to ensure that the project is meeting its intended goals and to inform continuous improvement efforts.

### Evaluation Plan

Using the logic model as a foundation, the advisory committee and development team identified key areas that required evaluation. They prioritized metrics that would provide actionable insights for improving the app and its integration into existing campus mental health and wellness services. Drawing on their collective expertise, the committee discussed various data collection methods. They aimed for a mixed methods approach to capture both quantitative and qualitative data, ensuring a comprehensive evaluation. Moreover, recognizing the evolving nature of digital health interventions, the committee built in mechanisms for ongoing review and refinement of both the app and the evaluation process itself.

Throughout this process, the committee emphasized the importance of flexibility and adaptability, acknowledging that the evaluation plan may need to be adjusted as the project progresses and new insights emerge. They also stressed the need for ongoing communication and collaboration among all stakeholders involved in the implementation and evaluation of the student mental health and wellness app. The evaluation questions that could address important outcomes are listed below.

1. To what extent does the use of the mobile app lead to reductions in symptoms of anxiety, depression, and other common mental health issues among students?
2. How many students download and actively use the app on a regular basis? What user characteristics or engagement patterns are associated with better outcomes?
3. How well is the app integrated with campus counseling, health, and other support services? What are the facilitators and barriers to this integration?
4. What are the key lessons learned from implementing the app, and what modifications or additional support are needed to sustain and scale the program in the long term?

Data collection methods will include pre-post surveys of app users to assess changes in knowledge regarding campus resources, presence and severity of mental health symptoms, emotional self-awareness, stigma and other barriers to care, confidence in and degree of help-seeking behaviors, and overall app usage data (downloads, login frequency, feature engagement). We also plan to conduct interviews and focus groups with app users, campus mental health staff, and other relevant stakeholders.

For continuous quality improvement, we plan to regularly review the evaluation results with the project team to identify the areas for app refinement and programmatic enhancements and use data to secure continued funding and support for sustaining and scaling the app-based program.

This evaluation plan (Table 1) outlines a mixed methods approach to assess the student mental health and wellness app project on multiple levels—individual student outcomes, community reach and usage, operational feasibility, and long-term sustainability. The plan emphasizes the importance of utilizing evaluation findings to drive ongoing improvement and broader dissemination of effective practices. Rigorous data collection, analysis, and reporting will be critical to demonstrating the value of this initiative to university stakeholders and external partners.

The evaluation team designed a process to evaluate cohorts of the students biannually. This process enables the team to monitor trends and changes at the population level across campus and assess the outreach, effectiveness, and impact of the app. In the first cohort, students who are new to the campus (ie, freshman, transfer, and first-year medical students) will be invited to enroll in the assessments. They will be required to provide informed consent before participating and will be compensated for their time and engagement in the quantitative and qualitative assessments (Multimedia Appendix 3).

**Table 1:** A summary of the evaluation plan for the mental health and wellness app (Willow).

Evaluation domains	Outcomes	Key performance indicators	Data sources and collection method
Campus services awareness, accessibility, and utilization	Improving access to mental health resources, improved efficiency of use of resources for individual psychological counseling, referral to the app by counseling and psychological services & health and wellness providers, improved utilization of on-campus services	Satisfaction, in-app services usage, Counseling and Psychological Services usage, effectiveness and relevance of the app, utilization of campus services	App analytics, in-app rating systems, in-app surveys and questionnaires, focus groups; interviews with counseling and psychological services & health and wellness providers
Student mental health	Reducing stigma, increasing peer support, improving mental health, improving emotional self-awareness, decreasing loneliness	Trend of improved score in mental health surveys, loneliness score, social connectedness on campus	App analytics, in-app surveys and questionnaires, focus groups
Serious mental illness	Detecting and predicting serious mental illness	Detect serious mental illness	App analytics, in-app surveys, and questionnaires
Student engagement and provider engagement	Increasing engagement with the app, increased use of features and services provided by the app, increased levels of awareness and utilization of the variety of resources and support available	Adoption rates, number of users, frequency of use, duration of use sessions, referral rates from providers	App analytics, in-app surveys and questionnaires, focus groups (students/providers)
App/platform usability and effectiveness	Continued development of app roadmap and milestones, plan for long-term sustainability to support continued deployment and improvement of the app	Daily, weekly, monthly active users, satisfaction rates, acceptability, and user-friendliness scores	In-app rating systems, in-app surveys and questionnaires to collect feedback, focus groups

### Ethical Considerations

This evaluation plan, which is described in more detail below, is approved by the UCSD institutional review board (approval 810254). Data will be collected using unique participant ID numbers that are based on the order of completion and will not be connected to any identifiable information about individual users. The app contains a digital consent form that participants complete before starting study activities. They receive an emailed copy and can access a video walk-through of the consent process. With rolling recruitment, participants can take time to decide and consult with the study team. Focus group members receive individual consent forms after eligibility confirmation. All consent forms are stored on a secure UCSD server accessible only to approved study coordinators. The consent mentions that participants can opt out at any time, and their deidentified data might be used for other studies without their consent. Participants have the opportunity to earn a maximum of US $25 per quarter for completing the surveys. Participants who take part in the focus groups will receive US $60. All incentives will be in the form of digitally delivered Amazon gift cards.

### Process and Participants

A marketing campaign was initiated to advertise the Willo app across the campus during the first quarter of the academic year 2024-2025. The campaign included multiple channels to reach out to students so that they can download and start using the app. Participants who agree to join the research portion of the app launch will provide electronic consent within the app. All participants will be asked to complete web-based assessments through Qualtrics at baseline fall quarter of 2024 and spring quarter of 2025. Additionally, we will conduct 6 focus groups each consisting of 10 students to gather qualitative data about their feedback and experience with the app. If participants consent, they may be contacted up to twice per calendar year until graduation or departure from UCSD.

### Measures

#### Metrics

Metrics to assess the evaluation domains are described below in more detail. We will use several tools and methodologies to collect data, including web-based surveys and in-app data for quantitative and focus groups and interviews for qualitative data. We based much of our approach on that used by the U-Flourish program [1,5] particularly in areas such as the longitudinal assessment of student well-being and the importance of a whole-campus approach to mental health. This collaboration helped us select a suite of evaluation measures that was not only scientifically validated but also of proven use in a large number of college students. This will also enable us to compare some of our findings to others in the U-Flourish network.

#### Surveys and Questionnaires

Validated mental health screening tools (eg, Generalized Anxiety Disorder Scale-7 items, Patient Health Questionnaire-8 items) and health behavior tools (eg, Sleep Condition Indicator-8 items, College Student Subjective Well-being Questionnaire) to measure changes in symptoms over time will be administered (Table 2). We also plan to include custom survey questions to gather user feedback and measure help-seeking behaviors such as their knowledge and interest of student resources and activities.

**Table 2:** List of questionnaires used in the evaluation of the app.

Label	Measure
Resources	Knowledge and Usage of Student Resources
GADS-7	Generalized Anxiety Disorder Scale-7 items
PHQ-8	Patient Health Questionnaire-8 items
WEMWBS-7	Warwick-Edinburgh Mental Wellbeing Scale-7 items
SCI-8	Sleep Condition Indicator-8 items
CSSWQ	College Student Subjective Wellbeing Questionnaire
ULS 4	UCLA Loneliness Scale-4 items
PSS-4	Perceived Stress Scale-4 items
SCOFF	Eating Disorder Diagnostic Scale
GBS	General Belongingness Scale
SCS-SF	Self-Compassion Scale-Short Form
RRS-B	Ruminative Response Scale-Brooding subscale
BCC	Barriers to Care Checklist
BACE-3	Barriers to Care Evaluation
CECA	Childhood Experiences of Care and Abuse
GLTEQ	Godin Leisure-Time Exercise Questionnaire
MSCS	Mindful Self-Care Scale-Mindful Relaxation Subscale
DERS-18	Difficulties in Emotional Regulation Scale

#### App Usage Analytics

At the aggregate level, measures such as weekly and monthly active users, average session duration, and most accessed resources will be tracked to monitor overall app engagement. For each individual user, the app will track their activities such as clicks, pages/resources browsed, events liked, and searched queries. These interactions will also be contextualized by individual app usage sessions. These metrics will help the app learn more about user preferences in general as well as their behaviors with respect to time and further allow the system to define and execute just-in-time adaptive interventions in the future by using varied notifications.

#### Interviews and Focus Groups

We plan to conduct semistructured interviews and focus groups with app users to gather qualitative feedback on their experiences and to understand the barriers, motivations, and perceptions about the app.

### Data Analysis and Interpretation

#### Quantitative Analyses

Quantitative data will be analyzed using appropriate statistical software (SPSS or similar) to determine if users are gaining knowledge about the featured services and resources and if the services and resources that are being highlighted are matching with the desires of the target populations.

To assess changes in mental health and wellness outcomes within and between cohorts over time, we will conduct repeated measures analysis of variance and mixed-effects modeling. These approaches are well-suited for analyzing longitudinal data with repeated observations per participant. The primary dependent variables will include standardized scores from validated instruments measuring mental health and well-being. Independent variables will include cohort membership, time (semester), usage of the Willo app, and engagement with university support services. Interaction terms (eg, time × app usage) will be included to assess potential moderating effects.

A power analysis was conducted using a conservative estimate of small-to-moderate effect sizes, consistent with those commonly reported in similar longitudinal mental health studies. With 850 participants per cohort and repeated measurements across 8 time points, the study is adequately powered (≥80%) to detect meaningful changes in outcomes over time and between groups, assuming an α level of .05.

#### Qualitative Analyses

We will employ Rapid Qualitative Analysis drawing on the structured and pragmatic methodology [30,31], which is particularly well-suited to applied health and implementation research settings where timely yet rigorous analysis is needed. Rapid Qualitative Analysis involves structured data reduction by using summary templates and matrix-based analysis to support both cross-case comparison and theme identification while maintaining methodological transparency and feasibility. To enhance trustworthiness, we will implement the following strategies.

1. Credibility: analyst triangulation and regular team-based discussions will be used to verify interpretations.
2. Dependability: a documented audit trail will capture analytic decisions and changes throughout the study.
3. Confirmability: independent checks of data summaries will ensure interpretive consistency.
4. Transferability: contextual information will be included to support the applicability of findings beyond the sample.

We will incorporate reflexivity throughout the analytic process, as recommended in reflexive approaches to qualitative research. Analysts will document positionality, and the team will engage in critical discussions about how their perspectives shape interpretation. In doing so, we also align with the call for “knowing practice” [32] in thematic analysis—owning our theoretical stance, making reflexive choices explicit, and avoiding the uncritical mixing of incompatible analytic paradigms.

## Results

As of March 2025, we are in the process of data collection. Preliminary data that have been collected include >600 responses to surveys from freshman, undergraduate transfer, and medical students. A second round of survey assessments to measure change is expected in April to May 2025. We have also conducted 2 focus groups with undergraduate freshman to date. Two focus groups with undergraduate transfer students and one with medical students are planned for April-May 2025. No data analyses have been performed as of yet. These data will be the baseline for the metrics used, and we plan to repeat the data collection with every cohort biannually.

## Discussion

The rising prevalence of mental health challenges among college students underscores the critical importance of addressing student mental health and wellness in higher education settings [33]. This project’s multifaceted approach, combining digital technology, user-centered design, and evidence-based interventions, represents a promising step toward improving access to mental health resources and support for university students.

By leveraging mobile app technology and potentially incorporating artificial intelligence–driven features, this initiative aligns with the increasingly tech-savvy nature of today’s student population. Knowing the rapid evolution of digital technologies, we tried to emphasize iterative modeling and personalized interventions in this app to effectively address student’s wellness challenges [27]. As digital natives, current and incoming university students are likely to find app-based mental health support more approachable and accessible than traditional services alone. This technological approach not only meets students where they are but also has the potential to reduce the stigma associated with seeking help—a significant barrier to mental health care utilization among young adults [34].

A key rationale for the potential effectiveness of this app lies in its ability to address one of the most critical barriers to student mental health support: timely access to resources and information. By providing a readily accessible platform that offers immediate information about available resources and services, the app has the potential to bridge the gap between students’ needs and appropriate support systems. This proactive approach is supported by research indicating that timely access to mental health resources is associated with better academic performance, higher retention rates, and improved mental health outcomes and overall well-being among university students [35,36].

The user-centered design process employed in this project is a key strength, ensuring that the final product is tailored to the specific needs and preferences of university students. By involving students at every stage, from the initial gap analysis to feature selection and usability testing, the app is more likely to resonate with its intended users and achieve higher engagement rates. This approach aligns with best practices in digital health intervention development and increases the likelihood of the app’s effectiveness and sustained use [37,38].

The integration of the mental health app with the campus student electronic medical record represents a significant advancement in coordinated care for college students. This linkage allows for a more comprehensive and holistic approach to student health and wellness, enabling seamless coordination between digital interventions and traditional health care services. The long-term objective of leveraging data from both the app and electronic medical record to improve individual and population health for the entire campus is particularly promising. This data integration could enable sophisticated predictive analytics and population health management strategies, allowing for the early identification of at-risk students and the development of targeted preventive interventions [39].

The potential for scalability to other college campuses is another significant aspect of this project. By designing the app with scalability in mind, there is an opportunity to extend its reach and impact across different student populations, potentially benefiting hundreds of thousands of students. This scalability also offers the possibility of gathering large-scale data on student mental health trends and intervention effectiveness, which could inform future research and policy decisions [40].

The interdisciplinary nature of the project, bringing together expertise from software engineering, public health, mental health and wellness, and design, is a testament to the complex, multifaceted nature of student mental health and well-being challenges. This collaborative approach ensures that the app is not only technologically sound but also grounded in public health principles and current best practices in mental health and wellness care. The data generated from this project could serve as a rich resource for researchers across these disciplines, potentially spurring new collaborations and lines of inquiry. For example, public health researchers might collaborate with artificial intelligence specialists to develop predictive models for mental health trends on university campuses.

It should be noted that, as with any digital health intervention, issues of data privacy, security, and ethical use of artificial intelligence (if implemented) must be carefully considered and addressed [41]. Ongoing evaluation and refinement of the app based on user feedback and outcome data will be crucial to ensuring its long-term effectiveness and relevance.

In conclusion, this project represents an innovative, student-centered approach to addressing the pressing issue of college student mental health. By combining technological innovation with evidence-based practices and interdisciplinary collaboration, it has the potential to significantly improve access to mental health support for university students. As the project moves forward, continued emphasis on user engagement, privacy protection, and integration with existing campus services will be key to its success and potential for broader impact across the University of California system and other institutions of higher education [42].

We plan to disseminate the findings and lessons learned from this project through peer-reviewed publications and multimedia channels, including social media, news, university websites, and conference presentations.

## Appendix

Multimedia Appendix 1 SPIRIT (Standard Protocol Items: Recommendations for Interventional Trials) checklist for protocols.

Multimedia Appendix 2 Focus group interview guideline.

Multimedia Appendix 3 Consent form.

## Abbreviations

SPIRIT: Standard Protocol Items: Recommendations for Interventional Trials
UCSD: University of California San Diego

## Data Availability

Data sharing is not applicable to this study, as no data sets were generated or analyzed during this study.

## References

[1] Duffy A, Saunders KEA, Malhi GS, Patten S, Cipriani A, McNevin SH, MacDonald E, Geddes J (2019). Mental health care for university students: a way forward?. The Lancet Psychiatry.

[2] Auerbach RP, Mortier P, Bruffaerts R, Alonso J, Benjet C, Cuijpers P, Demyttenaere K, Ebert DD, Green JG, Hasking P, Murray E, Nock MK, Pinder-Amaker S, Sampson NA, Stein DJ, Vilagut G, Zaslavsky AM, Kessler RC, WHO WMH-ICS Collaborators (2018). WHO World Mental Health Surveys International College Student Project: prevalence and distribution of mental disorders. J Abnorm Psychol.

[3] Pedrelli P, Nyer M, Yeung A, Zulauf C, Wilens T (2015). College students: mental health problems and treatment considerations. Acad Psychiatry.

[4] American College Health Association (2009). American College Health Association-National College Health Assessment Spring 2008 Reference Group Data Report (Abridged). J Americ Coll Health.

[5] Goodday SM, Rivera D, Foran H, King N, Milanovic M, Keown-Stoneman CD, Horrocks J, Tetzlaff E, Bowie CR, Pickett W, Harkness K, Saunders KE, Cunningham S, McNevin S, Duffy A (2019). U-Flourish university students well-being and academic success longitudinal study: a study protocol. BMJ Open.

[6] Liu CH, Stevens C, Wong SH, Yasui M, Chen JA (2018). The prevalence and predictors of mental health diagnoses and suicide among U.S. college students: implications for addressing disparities in service use. Depress Anxiety.

[7] Lipson SK, Lattie EG, Eisenberg D (2019). Increased rates of mental health service utilization by US college students: 10-year population-level trends (2007-2017). Psychiatr Serv.

[8] Duffy ME, Twenge JM, Joiner TE (2019). Trends in mood and anxiety symptoms and suicide-related outcomes among US undergraduates, 2007-2018: evidence from two national surveys. J Adolesc Health.

[9] King N, Pickett W, Rivera D, Byun J, Li M, Cunningham S, Duffy A (2023). The impact of the COVID-19 pandemic on the mental health of first-year undergraduate students studying at a major Canadian University: a successive cohort study. Can J Psychiatry.

[10] Oswalt SB, Lederer AM, Chestnut-Steich K, Day C, Halbritter A, Ortiz D (2020). Trends in college students' mental health diagnoses and utilization of services, 2009-2015. J Am Coll Health.

[11] Czyz EK, Horwitz AG, Eisenberg D, Kramer A, King CA (2013). Self-reported barriers to professional help seeking among college students at elevated risk for suicide. J Am Coll Health.

[12] King N, Pickett W, McNevin SH, Bowie CR, Rivera D, Keown-Stoneman Charlie, Harkness K, Cunningham S, Milanovic M, Saunders KEA, Goodday S, Duffy A, U-Flourish Student Well-BeingAcademic Success Research Group (2021). Mental health need of students at entry to university: baseline findings from the U-Flourish student well-being and academic success study. Early Interv Psychiatry.

[13] (2019). The Association for University and College Counseling Center Directors annual survey 2018. Association for University and College Counseling Center Directors.

[14] McGorry PD, Mei C, Dalal N, Alvarez-Jimenez M, Blakemore S, Browne V, Dooley B, Hickie IB, Jones PB, McDaid D, Mihalopoulos C, Wood SJ, El Azzouzi FA, Fazio J, Gow E, Hanjabam S, Hayes A, Morris A, Pang E, Paramasivam K, Quagliato Nogueira I, Tan J, Adelsheim S, Broome MR, Cannon M, Chanen AM, Chen EYH, Danese A, Davis M, Ford T, Gonsalves PP, Hamilton MP, Henderson J, John A, Kay-Lambkin F, Le LK, Kieling C, Mac Dhonnagáin Niall, Malla A, Nieman DH, Rickwood D, Robinson J, Shah JL, Singh S, Soosay I, Tee K, Twenge J, Valmaggia L, van Amelsvoort T, Verma S, Wilson J, Yung A, Iyer SN, Killackey E (2024). The Lancet Psychiatry Commission on youth mental health. Lancet Psychiatry.

[15] King N, Linden B, Cunningham S, Rivera D, Rose J, Wagner N, Mulder J, Adams M, Baxter R, Duffy A (2022). The feasibility and effectiveness of a novel online mental health literacy course in supporting university student mental health: a pilot study. BMC Psychiatry.

[16] Lipson SK, Eisenberg D (2018). Mental health and academic attitudes and expectations in university populations: results from the healthy minds study. J Ment Health.

[17] Lipson SK, Zhou S, Abelson S, Heinze J, Jirsa M, Morigney J, Patterson A, Singh M, Eisenberg D (2022). Trends in college student mental health and help-seeking by race/ethnicity: findings from the national healthy minds study, 2013-2021. J Affect Disord.

[18] Bolinski F, Boumparis N, Kleiboer A, Cuijpers P, Ebert D, Riper H (2020). The effect of e-mental health interventions on academic performance in university and college students: a meta-analysis of randomized controlled trials. Internet Interv.

[19] Ferrari M, Allan S, Arnold C, Eleftheriadis D, Alvarez-Jimenez M, Gumley A, Gleeson JF (2022). Digital interventions for psychological well-being in university students: systematic review and meta-analysis. J Med Internet Res.

[20] Serrano-Ripoll MJ, Zamanillo-Campos R, Fiol-DeRoque MA, Castro A, Ricci-Cabello I (2022). Impact of smartphone app-based psychological interventions for reducing depressive symptoms in people with depression: systematic literature review and meta-analysis of randomized controlled trials. JMIR Mhealth Uhealth.

[21] Pankow K, King N, Li M, Byun J, Jugoon L, Rivera D, Dimitropoulos G, Patten S, Kingslake J, Keown-Stoneman Charles, Duffy A (2024). Acceptability and utility of digital well-being and mental health support for university students: a pilot study. Early Interv Psychiatry.

[22] Broglia E, Millings A, Barkham M (2019). Counseling with guided use of a mobile well-being app for students experiencing anxiety or depression: clinical outcomes of a feasibility trial embedded in a student counseling service. JMIR Mhealth Uhealth.

[23] Duffy A (2023). University student mental health: An important window of opportunity for prevention and early intervention. The Canadian Journal of Psychiatry.

[24] Lattie EG, Lipson SK, Eisenberg D (2019). Technology and college student mental health: challenges and opportunities. Front Psychiatry.

[25] Firth J, Torous J, Nicholas J, Carney R, Pratap A, Rosenbaum S, Sarris J (2017). The efficacy of smartphone-based mental health interventions for depressive symptoms: a meta-analysis of randomized controlled trials. World Psychiatry.

[26] Fitzpatrick S, Crenshaw AO, Donkin V, Collins A, Xiang A, Earle EA, Goenka K, Varma S, Bushe J, McFadden T, Librado A, Monson C (2024). We have spent time, money, and effort making self-help digital mental health interventions: is anyone going to come to the party?. J Med Internet Res.

[27] Patrick K, Hekler EB, Estrin D, Mohr DC, Riper H, Crane D, Godino J, Riley WT (2016). The pace of technologic change: implications for digital health behavior intervention research. Am J Prev Med.

[28] Chan A, Tetzlaff JM, Altman DG, Laupacis A, Gøtzsche Peter C, Krleža-Jerić K, Hróbjartsson Asbjørn, Mann H, Dickersin K, Berlin JA, Doré Caroline J, Parulekar WR, Summerskill WS, Groves T, Schulz KF, Sox HC, Rockhold FW, Rennie D, Moher D (2013). SPIRIT 2013 statement: defining standard protocol items for clinical trials. Ann Intern Med.

[29] Logic model development guide: using logic models to bring together planning, evaluation, and action. W K Kellogg Foundation.

[30] Hamilton AB, Finley EP (2020). Reprint of: qualitative methods in implementation research: an introduction. Psychiatry Res.

[31] Taylor B, Henshall C, Kenyon S, Litchfield I, Greenfield S (2018). Can rapid approaches to qualitative analysis deliver timely, valid findings to clinical leaders? A mixed methods study comparing rapid and thematic analysis. BMJ Open.

[32] Braun V, Clarke V (2023). Toward good practice in thematic analysis: avoiding common problems and be(com)ing a researcher. Int J Transgend Health.

[33] Lipson SK, Phillips MV, Winquist N, Eisenberg D, Lattie EG (2021). Mental health conditions among community college students: a national study of prevalence and use of treatment services. Psychiatr Serv.

[34] Lattie EG, Adkins EC, Winquist N, Stiles-Shields C, Wafford QE, Graham AK (2019). Digital mental health interventions for depression, anxiety, and enhancement of psychological well-being among college students: systematic review. J Med Internet Res.

[35] Conley CS, Durlak JA, Kirsch AC (2015). A meta-analysis of universal mental health prevention programs for higher education students. Prev Sci.

[36] Bruffaerts R, Mortier P, Auerbach RP, Alonso J, Hermosillo De la Torre AE, Cuijpers P, Demyttenaere K, Ebert DD, Green JG, Hasking P, Stein DJ, Ennis E, Nock MK, Pinder-Amaker S, Sampson NA, Vilagut G, Zaslavsky AM, Kessler RC, WHO WMH-ICS Collaborators (2019). Lifetime and 12-month treatment for mental disorders and suicidal thoughts and behaviors among first year college students. Int J Methods Psychiatr Res.

[37] Yardley L, Morrison L, Bradbury K, Muller I (2015). The person-based approach to intervention development: application to digital health-related behavior change interventions. J Med Internet Res.

[38] Biagianti B, Hidalgo-Mazzei D, Meyer N (2017). Developing digital interventions for people living with serious mental illness: perspectives from three mHealth studies. Evid Based Ment Health.

[39] Bonet L, Torous J, Arce D, Blanquer I, Sanjuán Julio (2021). ReMindCare, an app for daily clinical practice in patients with first episode psychosis: a pragmatic real-world study protocol. Early Interv Psychiatry.

[40] Wasil AR, Taylor ME, Franzen RE, Steinberg JS, DeRubeis RJ (2021). Promoting graduate student mental health during COVID-19: acceptability, feasibility, and perceived utility of an online single-session intervention. Front Psychol.

[41] Torous J, Bucci S, Bell IH, Kessing LV, Faurholt-Jepsen M, Whelan P, Carvalho AF, Keshavan M, Linardon J, Firth J (2021). The growing field of digital psychiatry: current evidence and the future of apps, social media, chatbots, and virtual reality. World Psychiatry.

[42] Rith-Najarian LR, Boustani MM, Chorpita BF (2019). A systematic review of prevention programs targeting depression, anxiety, and stress in university students. Journal of Affective Disorders.

