# Supplementary material for: A Human-Centered Approach for a Student Mental Health and Well-Being Mobile App: Protocol for Development, Implementation, and Evaluation
Source: JMIR Res Protoc. 2025 Jul 18;14:e68368. doi: 10.2196/68368 (PMC12317289; doi:10.2196/68368)
Supplement: Multimedia Appendix 2 [file resprot_v14i1e68368_app2.docx]

**Focus Group Questions Outline**

**Session Set up: 10 min**

Facilitators will introduce themselves and remind people about the process and that they are going to be recorded but everything will be deidentified and analyses will occur from transcription. Facilitators will remind everyone to please use their identifiers to make a point whenever possible (i.e. participant numbers or colors or other)

Facilitators will ask individuals to go around the room and introduce themselves **before recording has begun** to get the group warmed up and ready to communicate with each other.

App Qualitative Focus Group Questions

**Icebreakers—10 minutes**

- Favorite things about San Diego
- An app they use regularly and why they like it
  - Could be a game, or podcast source, or anything like that.
  - *If there is a one or two items that get repeated by several participants or there is “me too” responses: What about that game/podcast/etc. do you particularly like?*
- When you first heard about the app, what did you hope it would have
  - *What kind of app did you think it would be based on the name and the location where you got it?*

**Usability—20-30 minutes**

- What did you think of the interface that the app offered.
  - *Was it easy to use? How did you like the look? Was there a spot that you particularly liked? Was there anything that made you want to come back? Did you use it to go to any partner sites (if that feature is available in this app version)?*
- How was navigating through the app. Were you able to locate specific resource you desired/required?
  - *Were they easy to find? What did you use the app for?* *Were resources located where you expected them to be? Did anything seem out of place? What additional resources do you think you might want on the app?*
- Did you experience any glitches or frustrations?
  - *Did anything feel intrusive? Was there anything that you wanted to do on the app that you weren’t able to do?*
- Does anyone have anything they want to add about the app itself?

**Acceptability-20-30**

- How relevant is this app to your daily needs?
- What changes you’d like to see in the app to makes it worth using compared to alternatives?
- How well does the app addresses ones specific challenges?

What barriers might prevent people from using this app?

**Mental Health—20-30 minutes**

- What did you think of the various tools available on the application in regard to mental health improvement and maintenance?
  - *Follow up with specifics from the app itself to see if they used any of the tools that on the app.*
- What main strengths or weaknesses did you notice in the app?
  - *Was there a spot or offering that you particularly liked? Was there anything that made you want to come back? Did anything feel intrusive?*
- Was there anything you wish the app could have addressed regarding mental health?
- How did using this app change your relationship with or awareness of your mental health, if at all?
- If you have used any other mental health apps before, how did this compare?
  - *What did others have that you wish this one did?*
- How should apps balance supporting the user and maintaining privacy and personal boundaries?
- What features or strategies could help encourage regular utilization of a mental health app like this?
- How could an app foster a sense of belonging and security in a community of users while maintaining a safe and supportive atmosphere?
- How do you think you will use this app in your mental health journey moving forward?
- Does anyone have anything they want to add?

**Wrap Up—20 minutes**

- Facilitator to provide a brief summary of key observations from notes for each section and ask if anyone would like to share anything that has occurred to them since we were last talking about those elements
- **If time:** Facilitator to provide a brief summary of key observations from notes **from other focus groups** and ask if anyone has any thoughts on those observations.
