# Supplementary material for: A Human-Centered Approach for a Student Mental Health and Well-Being Mobile App: Protocol for Development, Implementation, and Evaluation
Source: JMIR Res Protoc. 2025 Jul 18;14:e68368. doi: 10.2196/68368 (PMC12317289; doi:10.2196/68368)
Supplement: Multimedia Appendix 3 [file resprot_v14i1e68368_app3.pdf]

UNIVERSITY OF CALIFORNIA, SAN DIEGO
CONSENT TO PARTICIPATE IN RESEARCH

1. Study Title and Number

Title: WILLO: Student Health and Wellness
Study # 810254

2. Principal Investigator

Job Godino, MS, PhD
Assistant Professor in the Department of Family Medicine and Public Health at UCSD and the Director of Applied Research and Technology at EPARC

3. Principal Investigator Phone Number, Research Team Number, and Emergency Contact Number

EPARC Research Team Number: (858) 534-9315

4. Study Sponsor

UC San Diego is paying to conduct this research study, with funds from the Center for Health Innovation.

5. Study Overview

This research study is being conducted because the challenges regarding health among college students are multifaceted and can stem from various factors, including academic pressure, perceived lack of time to engage in healthy behaviors, social expectations, financial stress, homesickness, relationship issues, and the overall transition to independent living.

Addressing these issues requires a multifaceted approach involving proactive mental health education, destigmatization of seeking help, improving access to counseling services, implementing stress reduction programs, and fostering a supportive campus environment. Luckily, UCSD has many services designed to address these, and other, elements of health. Creating awareness about self-care strategies, encouraging open conversations about health, and ensuring that students are aware of what is available and can access services and events with ease can empower students to prioritize their well-being and seek assistance when needed.

We are inviting you to participate in a research study because you are a student at UCSD, and we want to know more about the usability and acceptability of an app that can provide personalized recommendations for activities and resources that will help to maintain and enhance the health of college students.

This form explains the research so that you may make an informed decision about participating.

- Research is voluntary - whether or not you participate is your decision. You can discuss your decision with others (such as family, friends or another physician).
- You can say yes, but change your mind later.
- If you say no, we will not hold your decision against you.
- You can say no even if the person inviting you is part of your healthcare team.
- Your decision will not affect your health care or other benefits you may be entitled to.
- Please ask the study doctor or study team questions about anything that is not clear, and feel free to ask questions and mention concerns before, during, and after the research.
- You may consult with friends, family, a personal doctor, or anyone else before deciding whether or not to be in the study.

Protocol #810254 | v8 | Approved: Jul 16, 2024

- You will be given a copy of this consent form and the Participant's Bill of Rights.

The purpose of this research study is to gather data about an application that will be considered standard of care for incoming students. The app aims to provide opportunities to better connect students with university resources that will be tailored to their needs and interests while also providing a model for stepped care ranging from building of resilience through self-monitoring to support for seeking and scheduling treatments for health concerns. We want to emphasize that **you can get the app for free and use it for free without participating in this research study.**

The most common risks or discomforts of this study are some feelings and thoughts that may come up when answering questions about your own health and wellness. If these become overwhelming, please refer to the resources on the app. You do not have to answer any questions that make you uncomfortable. A complete listing of possible risks and discomforts associated with this study can be found in Section 9 of this document.

The alternative to being in this study is not to participate.

More detailed information about this research study is provided below.

6. Whom can I talk to if I have questions?

If during your participation in the study you have questions or concerns, or if you think the research has hurt you, contact the research team at the numbers listed in Section 3 on the first page of this form. You should not agree to participate in this study until the research team has answered any questions you have about the study, including information contained in this form.

If before or during your participation in the study you have questions about your rights as a research participant, or you want to talk to someone outside the research team, please contact:

- UC San Diego Office of IRB Administration at 858-246-4777 or irb@ucsd.edu

7. How many people will take part?

We plan to enroll 850 people here. The research will include 400 incoming freshman students, 400 newly transferred students, and 50 new medical students.

8. What happens if I take part in the research?

Here is what will happen if you agree to be in this study:

You will first be evaluated for eligibility to be in this study. If you are eligible, and you agree to participate, you will complete this consent form, Please note that this consent form is different from the terms of service that you will be asked to complete when you download the app regardless of your participation in this study. While it is separate from the study consent form, it will be required to download the app, and therefore be eligible for the study.

In addition to the basic questionnaire that everyone who downloads the application will complete, we will be asking you to complete several surveys upon enrollment in this research component of this application rollout ("baseline") and then again at the beginning of the Winter and again at the beginning of the Spring quarters. These will include questions about your knowledge of student resources and activities, and your interest in learning about more of them.

Protocol #810254 | v8 | Approved: Jul 16, 2024

You will also be given the opportunity to complete an additional set of surveys about your own health and wellness. These will be divided into 5 key surveys (set 2A), and then an additional 23 surveys (set 2B). You do not have to complete either of the second set of surveys to participate in the study, but you will receive extra compensation if you complete both sets.
The questions about student resources and activities will ask you about your knowledge of, and interest in, a number of different groups and activities that are available here on campus.
The health and wellness surveys will ask you questions about your levels of anxiety, depression, social connectedness, financial security, and other potentially sensitive items.
We expect that the first batch of surveys (about campus resources and your knowledge of them) will take 15-30 minutes to complete.
We expect that the 5 key surveys regarding your health and wellness will take 1525 minutes to complete.
We expect that the final 13 surveys will take 45-60 additional minutes.
These surveys do not all have to be completed at one time and can instead be completed over a three day period.
You do NOT have to complete the health and wellness surveys to complete the campus resource surveys but do have to complete the campus resource surveys to complete the health and wellness surveys.

If you average more than 8 hours of use in the app during the first 30-days after you download the app, and are selected by the study team, you may be asked to participate in a focus group.
NOTE: You will be invited but not required to participate. In the event that you do agree to participate you will be asked to sign an additional informed consent. At that time we will go into more detail about the expectations of participating, but we have also given some brief details here.
If you are chosen and agree to participate you will be asked open-ended questions about your experience with the app. The focus groups will be made up of 20 participants from each cohort (two groups of ten), and 20 participants from all cohorts that specifically interacted with the mental health and wellness content (two groups of ten). It will involve open-ended questions about your experience with the app and the study in general. The focus group is completely optional and does not affect one's participation abilities in the study at large.

You will also have the option to extend your participation longer and agree to be contacted up to three times a year (at the beginning of Fall, Winter, and Spring quarters) until you leave UCSD to ask about your experience with updated versions of the app. This longer term follow up option would involve ongoing questions about your experiences with the app, and about your health and wellness
<u>Study Schedule:</u>
Group 1: All participants
Group 2: Agree to mental health survey
Group 3: Agree to mental health survey and engage with the app >8 hours in the first 30 days of use.

Timepoint	Usability Questions	Campus Resource Survey	Mental Health/Wellbeing Questions
Baseline (Fall Quarter)	1,2,3	1,2,3	2,3
First Follow up (Winter Quarter)	1,2,3	1,2,3	2,3
Second Follow up (Spring)	1,2	1,2	2
Longer Term Follow Up (optional)		1,2	1,2

As you read this form, ask questions if something is not clear.

9. What are the risks and possible discomforts?

Participation in this study may involve risks or discomforts. We will minimize the possibility of results from this research being linked to you, but there is always the remote possibility that information from the research may be disclosed.

Protocol #810254 | v8 | Approved: Jul 16, 2024

Risks of Loss of Confidential Information: There is also a risk that information about you could be released to an unauthorized party. To minimize this risk, we will use only the highest security data collection and storage options available at UCSD and use a code on any information we collect and we will keep a link between the code and your identity in a different location. If you are invited and choose to participate in the focus group, please know that although we ask everyone in the group to respect the privacy and confidentiality of participants, and to keep the discussion in the group confidential, we cannot guarantee this. Please keep this in mind when choosing what to share in the group setting.

Risks of Collection of Sensitive Information: Some of the questions we will ask you are personal. You may feel embarrassed or stressed. You may ask to see the questions before deciding whether or not to take part in this study.

Risks of Interviews/Questionnaires/Quality of Life Assessments that Discuss Sensitive Issues: Some of these questions may seem very personal or embarrassing. They may upset you. You may skip any question that you do not want to answer. If the questions make you very upset, we will help you to find a counselor, refer you to an appropriate clinic for follow up, or you can contact the UCSD Triton Team for immediate on-campus assistance.

Possible Unknown Risks: In addition, there might be risks that we cannot predict at this time. You will be informed of any new findings that might affect your health or welfare, or might affect your willingness to continue in the research.

If our surveys indicate that you are at acute mental health risk, we will reach out with recommendations for appropriate care.

10. How will information about me be protected?

While we cannot guarantee complete confidentiality, we will limit access to information about you. Only people who have a need to review your information, documents, or specimens will have access. These people might include:

- Members of the research team and other staff or representatives of UCSD whose work is related to the research or to protecting your rights and safety.
- Representatives of the study sponsor or product manufacturer
- Representatives of Federal and other regulatory agencies who make sure the study is done properly and that your rights and safety are protected.

Study information will be labeled with a code instead of your name or other information that can easily identify you. The record linking your identifying information (name, address, etc.) and the code will be kept separate from the rest of the study information.

The results of this study may be published once the study is completed. However, we will keep your name and other identifying information confidential. We expect this study will be completed in 4. This is only an estimate and the actual time to complete the study may be longer or shorter depending on a number of factors.

11. Will I need to pay to participate in the research?

There will be no cost to you for participating in this study.

12. What if I agree to participate, but change my mind later?

Protocol #810254 | v8 | Approved: Jul 16, 2024

You can stop participating at any time for any reason, and it will not be held against you. Your choice will not affect any treatment relationship you have with healthcare providers at UC San Diego Health or any services you receive from them. No matter what you decide, there will be no penalty to you. You will not lose medical care or any legal rights.

If you stop early, please contact us immediately so that we may update your status and list a reason for research purposes.

If you stop participating, we may not be able to remove the information we have already collected about you or specimens we have already collected from you.

13. What will happen to information and/or biospecimens collected from me?

The data we collect with your identifiable information (for example, your name, medical record number, or date of birth) as a part of this study may be used to answer other research questions or may be shared with other investigators for other research. If we do so, we will remove all identifiable information before use or sharing. Once identifiers have been removed, we will not ask for your consent for the use or sharing of your data in other research.

While your privacy and confidentiality are very important to us and we will use safety measures to protect it, we cannot guarantee that your identity will never become known.

14. What are my responsibilities if I take part in this research?

If you take part in this research, you will be responsible for at the minimum completing the first surveys about your experience with the app and your knowledge of resources on campus. You are encouraged to also complete the mental health and wellness surveys, and participate in the focus group if invited, and/or agree to longer term follow-up, but those are optional. **There is no minimum amount of time you need to spend on this app, but we would prefer that you interact with it for at least 8 hours over the course of your study participation.**

15. Will I be compensated for participating in the research?

If you agree to take part in this research, we will provide you with Amazon digital gift cards for your time and effort. See below for compensation schedule:

$10 for completing the baseline surveys regarding campus resources
$5 for completing the first 5 health and wellness surveys at baseline
$10 for completing the additional 13 health and wellness surveys at baseline

You will receive this same level of compensation at each of the follow up assessments based. Specifically, you will receive
$10 for completing surveys regarding campus resources
$5 for completing the first 5 health and wellness surveys
$10 for completing the additional 13 health and wellness surveys
You are specifically agreeing to be contacted again **in Winter and Spring quarter of this academic year.**
If you agree to be contacted for a longer period (see below) you will be compensated at least this amount of money for future assessments.

Although you will be asked to sign an additional consent form, and we will discuss the expectations and risks in more detail, if you use the app for >8 hours during the first 30 days following download you will be eligible for $60 for participating in the focus groups

Protocol #810254 | v8 | Approved: Jul 16, 2024

If you receive compensation in excess of $600 per calendar year, your name and Social Security number will be collected and released to the UC San Diego Office of Accounting to process the Form 1099-Misc for Internal Revenue Service (IRS) tax-reporting purposes.

16. What else is important for me to know?

All students are going to be invited to use the WILLO application, but only some students are being invited to be part of this research study. You do NOT have to be part of this research study to use this application.

17. What are my rights when providing electronic consent?

California law provides specific rights when you are asked to provide electronic consent:

- You have the right to obtain a copy of the consent document in a non-electronic format.
- You have the right to provide consent in a non-electronic format.
- If you change your mind about electronic consent, you have the right to request your electronic consent to be withdrawn and you can then provide consent in a non-electronic format; however, a copy of your electronic consent will be maintained for regulatory purposes. If you wish to withdraw your electronic consent please tell the study team.

This agreement for electronic consent applies only to your consent to participate in this research study.

18. Additional Choices to Consider

In Section 8, we described some extra procedures regarding being **contacted to participate in a focus group.** These extra procedures are optional, meaning that you can participate in the study even if you refuse the procedures. Please indicate your choice by initialing the appropriate line below:

___________I AGREE to be contacted to learn more about these optional procedures, if eligible.

___________I DO NOT AGREE be contacted to learn more about these optional procedures, if eligible.

In Section 8, we described some extra procedures regarding **being contacted up to twice a year in future academic years** until you leave UCSD to answer surveys. These extra procedures are optional, meaning that you can participate in the study even if you refuse the procedures. Please indicate your choice by initialing the appropriate line below:

___________I AGREE to be contacted to consider participating in these optional procedures.

___________I DO NOT AGREE to be contacted to consider participating in these optional procedures.

The study team would like your permission to contact you about participating in future studies. You may still join this study even if you do not permit future contact. You may also change your mind about this choice. Please initial your choice below:

___________YES, you may contact me

___________NO, you may NOT contact me

Protocol #810254 | v8 | Approved: Jul 16, 2024

Signature Block for Adults Able to Provide Consent

Participant

I have received a copy of this consent document and a copy of the "Experimental Participant's Bill of Rights" to keep. I agree to participate in the research described in this form.
Lastly, no enticements, benefits, or financial incentives were used at any level of the process to incentivize my abortion or the donation of Human Fetal Tissue.

Printed Name of Participant

Signature of Participant Date

Person Obtaining Consent

I document that:

- *I (or another member of the research team) have fully explained this research to the participant.*
- *I have personally evaluated the participant's understanding of the research and obtained their voluntary agreement.*

Printed Name of Person Obtaining Consent

Signature of Person Obtaining Consent Date

Witness (if applicable)

I document that the information in this form (and any other written information) was accurately explained to the participant. The participant appears to have understood and freely given consent to join the research.

Printed Name of Witness

Signature of Witness Date

Experimental Participant's Bill of Rights

Every individual asked to participate in a research study has the right to be:

Protocol #810254 | v8 | Approved: Jul 16, 2024

1. Informed about the nature and purpose of the study.

2. Provided an explanation of the procedures to be followed in the research study, and whether any of the drugs, devices, or procedures is different from what would be used in standard practice.

3. Given a description of any side effects, discomforts, or risks that you can reasonably expect to occur during the study.

4. Informed about any benefits that would reasonably be expected from the participation in the study, if applicable.

5. Informed about of any alternative procedures, drugs, or devices that might be helpful, and their risks and benefits compared to the proposed procedures, drugs or devices.

6. Told of the types of medical treatment, if any, available if complications should arise.

7. Provided an opportunity to ask any questions concerning the research study both before agreeing to participate and at any time during the course of the study.

8. Informed that individuals can refuse to participate in the research study. Participation is voluntary. Research participants may refuse to answer any question or discontinue their involvement at any time without penalty or loss of benefits to which they might otherwise be entitled. Their decision will not affect their right to receive the care they would receive if they were not in the experiment.

9. Provided a copy of the signed and dated written consent form and a copy of this form.

10. Given the opportunity to freely decide whether or not to consent to the research study without any force, coercion, or undue influence.

If you have any concerns or questions regarding the research study contact the researchers listed at the top of the consent form.

If you are unable to reach a member of the research team and have general questions, or you have concerns or complaints about the research study, research team, or questions about your rights as a research participant, please contact:

- UC San Diego Office of IRB Administration at irb@ucsd.edu or 858-246-4777

Protocol #810254 | v8 | Approved: Jul 16, 2024
